# Preact to lower the risk of falling by customized rehabilitation across Europe: the feasibility study protocol of the PRECISE project in Italy

**DOI:** 10.3389/fpubh.2024.1293621

**Published:** 2024-03-22

**Authors:** Flora D’Ambrosio, Michael Harbo, Danilo Contiero, Anna Rita Bonfigli, Diletta Cicconi, Niels Heuer, Arend Roos, Christian Fischer Pedersen, Paolo Fabbietti, Cristina Gagliardi

**Affiliations:** ^1^IRCCS INRCA, Ancona, Italy; ^2^DigiRehab A/S, Viborg, Denmark; ^3^ROOS Health, Middelburg, Netherlands; ^4^DigiRehab Benelux, B.V., Middelburg, Netherlands; ^5^Department of Electrical and Computer Engineering, Aarhus University, Aarhus, Denmark

**Keywords:** falls, rehabilitation, home training, smartphone app, aging, public health

## Abstract

**Introduction:**

Falls are a major worldwide health problem in older people. Several physical rehabilitation programs with home-based technologies, such as the online DigiRehab platform, have been successfully delivered. The PRECISE project combines personalized training delivered through the application with an artificial intelligence-based predictive model (AI-DSS platform) for fall risk assessment. This new system, called DigiRehab, will enable early identification of significant risk factors for falling and propose an individualized physical training plan to attend to these critical areas.

**Methods:**

The study will test the usability of the DigiRehab platform in generating personalized physical rehabilitation programs at home. Fifty older adults participants will be involved, 20 of them testing the beta version prototype, and 30 participants testing the updated version afterwards. The inclusion criteria will be age ≥65, independent ambulation, fall risk (Tinetti test), Mini Mental State Examination ≥24, home residents, familiarity with web applications, ability and willingness to sign informed consent. Exclusion criteria will be unstable clinical condition, severe visual and/or hearing impairment, severe impairment in Activities of Daily Living and absence of primary caregiver.

**Discussion:**

The first part of the screening consists in a structured questionnaire of 10 questions regarding the user’s limitations, including the risk of falling, while the second consists in 10 physical tests to assess the functional status. Based on the results, the program will help define the user’s individual profile upon which the DSS platform will rate the risk of falling and design the personalized exercise program to be carried out at home. All measures from the initial screening will be repeated and the results will be used to optimize the predictive algorithms in order to prepare the tool in its final version. For the usability assessment, the System Usability Scale will be administered. The follow-up will take place after the 12-week intervention at home. A semi-structured satisfaction questionnaire will also be administered to verify whether the project will meet the needs of older adults and their family caregiver.

**Conclusion:**

We expect that personalized training prescribed by DigiRehab platform could help to reduce the need for care in older adults subjects and the care burden.

**Clinical trial registration**: [https://clinicaltrials.gov/], identifier [NCT05846776].

## Introduction

Globally, falls are a major health problem worldwide. It is estimated that 684,000 fatal falls occur worldwide each year, making falls the second leading cause of unintentional death after road accidents (1).

Falls occur mainly in older people; the most affected age group is between 74 and 85 years (2). In Europe, on average 35,848 older adults people die each year as a result of a fall, and the number of known accidents is probably underestimated as compared to the number of real events (3).

Falls in the older adults are also related to the incidence and outcome of the injury, since among these people there is a high prevalence of comorbidities and other physiological changes associated with age, like the slowing of protective reflexes, which make even minor falls particularly dangerous. In addition, healing from a fracture is usually slower and lesser in older people, increasing the risk of subsequent falls and further complications (4).

Physical inactivity is one of the main causes that contribute to increase the risk of falling in the older adults, accelerating the decline of physiological functions with a negative impact on balance control, muscle strength and walking.

Fall prevention programs targeted at high-risk groups can help reduce injury rates by 20–40% (5). These activities are potentially cost-effective and can substantially reduce the healthcare costs associated with fall-related injuries. In particular, exercise programs aimed at muscle strengthening can significantly contribute in reducing the risk of falling in the older adults and preserving their functional independence and quality of life (6). The implementation of these programs also has a significant economic value since preventing falls can considerably reduce the high hospitalization costs related to them (for example as a result of bone fractures, thromboembolic events, heart attacks, etc.) and the need for long-term care.

For some time in the countries of northern Europe (for example Denmark and Norway), where home care for the older adults population is particularly widespread, physical rehabilitation programs with home-based technologies have been successfully tested (7).

An example is the DigiRehab platform,^1^ which aims to improve the physical and psychological condition of older adults home residents through personalized physical rehabilitation programs that can be used remotely.

In a study conducted by the National Association of Danish Municipalities (Kommunernes Landsforening) comparing 10 technology solutions with documented effect, it is concluded that in older adults people who took advantage of the DigiRehab home-based rehabilitation program this type of intervention has shown effective in improving the physical fitness of the older adults and reducing their care needs, with significant savings in the hours of home care provided by the municipality (8).

The PRECISE project takes the positive results achieved with the DigiRehab application in home rehabilitation and takes a further step in this direction by combining the personalized training delivered through the application with an artificial intelligence-based predictive model (AI-DSS platform) for fall risk assessment in the older adults.

This new system, called DigiPrehab, will enable early identification of the older adults with significant risk factors for falling and propose an individualized physical training plan to attend to the identified critical areas.

## Study design

A pre-post feasibility study will be conducted in older adults subjects to test the usability of an application for personalized rehabilitation exercises at home. Patients will undergo a questionnaire and physical assessment both at baseline and at the end of the 12-week intervention.

### Study aims

Primary and secondary outcomes as well as clinical assessment are described in Table 1.

**Table 1:** Outcomes and clinical assessment.

Primary outcome	Clinical assessment
Usability	System usability scale questionnaire
Acceptability	Semi-structured satisfaction questionnaire
Secondary outcome	Clinical assessment
Physical strength test	
Balance	Standing balance Dynamic balance
Lower limbs strength	30 s rise from sit position Long steps Push away Getting down and up with the support of a chair
Gait speed	Gait speed (4 m) Timed up and go
Need for help test	
	Structured questionnaire

The primary outcome will be the assessment of the usability of the system as well as its acceptability from users and caregivers.

Secondary outcomes will be variation in physical strength and limitations tests’ scores after the intervention.

### Settings

This study is being conducted at the Outpatients section of the Clinical Unit of Rehabilitation Medicine of IRCCS INRCA, Ancona, Italy.

### Participants

For the purposes of this pilot study, the first experience in using of this app in Italy has been planned to enroll a sample of older adults people in order to test the usability of the app, and its liking by this population. Fifty participants will be involved, 20 of them testing the beta version prototype of DSS, and 30 participants testing the updated version afterwards. The two groups will be different to avoid the implicit learning effect, but the enrolment criteria will be the same.

Inclusion criteria:

- 65 years of age or older;
- male and female;
- independent ambulation;
- fall risk assessed by Tinetti test score 19–24 (9);
- Mini Mental State Examination ≥24 (10);
- residents at home;
- familiarity with web applications;
- ability and willingness to sign informed consent.

Exclusion criteria:

- unstable clinical condition by judgment of the physician;
- severe visual and/or hearing impairment;
- severe impairment in physical activity (Activities of Daily Living scale <4) (11);
- absence of primary caregiver;

### Platform description

The DigiRehab platform is a digital exercise tool aimed at older adults citizens in need of homecare assistance. Developed by Danish physiotherapists it uses personally based exercise programs to increase the citizens’ self-reliance and reduce their need for homecare.

More than 15,000 Danish older adults have completed a DigiRehab program in Denmark, and in collaboration with Aarhus University this data has been used to develop both a Decision Support System based on AI-ML and a version of the screening that targets older adults citizens not yet in need of home care with a special focus on fall prevention. The Prehab screening is an early detection tool, designed to highlight on frail older adults and supply the opportunity to prevent preventable accidents from happening, by introducing targeted tailor-made strength training, at home.

### Patient recruitment

Patients will be selected by the outpatient department at the Clinical Unit of Rehabilitation Medicine of IRCCS INRCA, in Ancona. The subjects will be contacted to schedule a visit with the clinical team. Once the compliance with the inclusion and exclusion criteria of the study are verified and the informed consent is obtained, the team will proceed with the baseline evaluation.

No calculation of the sample size is foreseen as the study is configured as a pilot feasibility study which typically includes a limited number of participants (in our case 20 participants testing the beta version prototype of DSS and 30 the updated version).

## Methods

### Patient evaluation

At baseline, after the collection of demographic data, the physical strength test and the limitations test will be administered. The tests are administered through the DigiRehab platform and the results are sent to the Decision Support System Platform in order to assess the risk of falling using AI-ML. Moreover, the SF12 questionnaire for the evaluation of quality of life will be assessed.

**Figure 1:**
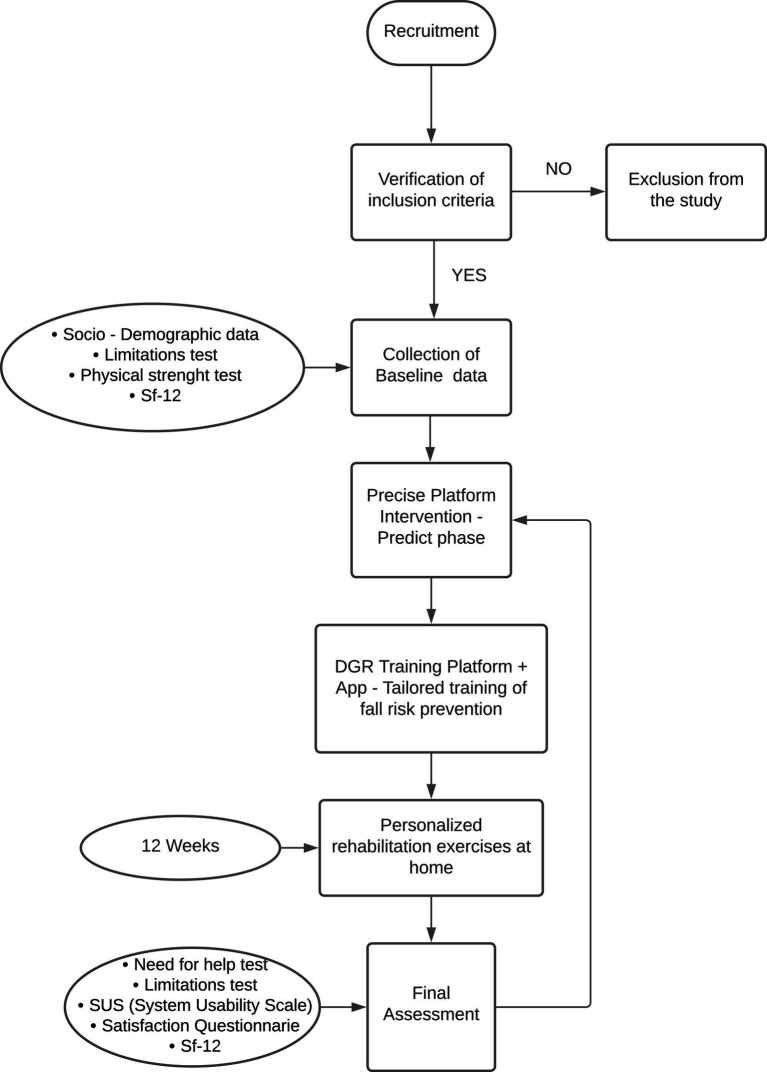
Flow chart of the PRECISE study.

At follow-up, patients’ evaluation will be assessed by means of the risk of falling screening (limitations test and physical strength test), and SF12, the SUS scale and a questionnaire of satisfaction (see Figure 1).

### Initial fall risk screening

The initial screening will be carried out by our rehabilitation specialists with expertise in fall risk prevention. The purpose of the screening will be to map the user’s functional capacity and to propose, using the AI-DSS algorithm developed by the project, a customized exercise program based on the user’s specific rehabilitation needs. The screening will take place in the presence and with the cooperation of the caregiver.

The screening consists of two parts: a structured questionnaire consisting of 10 questions regarding the user’s general limitations in daily life, named limitations test, and a second part including 10 physical tests aimed at assessing the functional status of the older adults person, named physical strength test (Table 1).

Specifically, the first part of the screening is based on a structured questionnaire containing 10 Likert scales with 5 response options (from no difficulty to high difficulty) regarding the following topics: (1) household chores, (2) personal care, (3) health, (4) nutrition, (5) sleep, (6) mobility, (7) dizziness, (8) fear of falling, (9) social interaction, and (10) vision of the future.

The physical strength screening, on the other hand, involves the following 10 exercises:

(1) 30 s rise from sit position test (from a chair);
(2) Standing balance (chair);
(3) Long steps (lunges);
(4) Four meters’ gait speed test Part 1 (Level);
(5) Four meters’ gait speed test Part 2 (Speed);
(6) Time up and go test;
(7) Dynamic balance;
(8) Push away;
(9) Getting down…
(10) …and up with the support of a chair.

Based on the initial screening, the user will receive two scores between 0 and 100, one indicating his limitations and one indicating his physical strength. These scores will help define the user’s individual profile on the basis of which the DSS platform will design the personalized exercise program to be carried out at home (Table 2).

**Table 2:** Initial fall risk screening.

Limitations	Physical strength
(1) General housekeeping	(1) 30″ rise from sit position test (from a chair)
(2) Personal care	(2) Standing balance (chair)
(3) Health	(3) Long steps (lunges)
(4) Nutrition	(4) Four meters’ gait speed test Part 1 (Level)
(5) Sleep	(5) Four meters’ gait speed test Part 2 (Speed)
(6) Mobility	(6) Time up and go test
(7) Dizziness	(7) Dynamic balance
(8) Concerned about falling	(8) Push away
(9) Maintain social contact	(9) Getting down…
(10) View of the future	(10) …and up with the support of a chair

### Assignment of personalized exercise program

Once the screening will be completed, using the algorithm, the app will assign to the participants a personalized exercise program, choosing among the following exercises:

- Squat at door: sit on chair in front of open door with hands on doorknobs; stand by pulling the doorknobs.
- Squat on chair: sit on chair with arms folded on the chest; stand up and sit down.
- Squat with knee-lift: sit on chair with arms folded on chest; stand-up, lift one knee to horizontal, lower knee and sit back down; stand up, lift other knee in the same way.
- Squat with heel-raise: stand in front of chair with arms folded on chest; lift on heel of base; bent knees still with one heel of base-if possible, sit down on chair; stand up and repeat with other heel of base.
- Stand-no support: stand behind a chair with hands on backrest; let go of backrest and keep balance as long as possible.
- Toe-raise with support: stand behind a chair with hands on backrest; support as little as possible when doing toe raises.
- Toe-raise: stand behind a chair not using it for support; lift alternately one leg and only use backrest for support if loosing balance.
- One leg balance: stand behind a chair not using it for support; lift alternately one leg and only use backrest for support if loosing balance.
- Weight-shift with support: stand supported by hand on backrest of a chair; step forward with one leg and shift weight to front leg; shift weight back to other leg.
- Weight-shift without support: step forward with one leg and shift weight to front leg; shift weight back to other leg.
- Lunge with support: stand supported by hand on backrest of a chair; step forward with one leg and shift weight to front leg; bent as much in front knee so back knee almost touches floor; get back up fast.
- Lunge: step forward with one leg and shift weight to front leg; bent as much in front knee so back knee almost touches floor; get back up fast.
- Step on book: stand in front of a thick book; step onto it with one foot then the other.
- Step over book: stand in front of a thick book; step onto it with one foot and the other foot across it.
- Step onto box or stair: stand in front of a box or stair; step onto it with one foot and then the other.
- Step over box: stand in front of a box or stair; step onto it with one foot and the other foot across it.
- Step forward-sideways: forward one step-and back; sideways one step and back; same with other leg.
- Knee raise: knee raises standing.
- Step forward-sideways-backwards: forward one step and back; sideways one step and back, backward one step and back; same with other leg.
- Knee to elbow: knee rises with opposite elbow meeting knee.
- Timed up and go: stand up from a chair and walk a 4 meters’ distance as fast as possible.

The algorithm will select from 5 to 8 exercises among the ones listed above; 2 or 3 sets of 8–12 repetitions should be performed for each exercise. During initial coaching, it is recommended to start with 2 sets of 8 repetitions and increase sets and repetitions going on. In addition, the system allows the coach to modulate the difficulty of the exercises according to each patient’s specific condition.

### Caregiver training session

Following the assignment of the specific program to the user, in the same session the caregiver will be instructed on the correct execution of the exercises and how to avoid any risks that might exist in relation to the user’s state of health.

On this occasion, each participant will receive free loan a tablet equipped with an internet connection for the duration of the trial, from which they will be able to follow the exercise program intended for them.

### On-going monitoring

One of our staff members with a degree in Adapted Preventive Exercise Activities, for the 12-week trial, will work alongside the Clinical Unit of Rehabilitation Medicine of IRCCS INRCA in order to assist the participants while performing the screening, explaining and demonstrating the exercises. At the same time, the specialist will remain available to answer any questions and/or requests from users, either on the performance of the exercises or on the use of the platform. In addition, every 2–4 weeks, the staff members will contact, by phone or email, some randomly selected participants to conduct spot checks on the performance of the exercises and adherence to the program.

### Follow-up evaluation

The follow-up survey will take place at the conclusion of the 12-week intervention. All measures from the initial screening will be repeated to check for any changes in the users’ condition. In particular, it will be noted whether the limitations score has decreased since the first survey and whether the physical strength score has increased instead. Development in both the over-all and the sub-scores will be used by the DigiRehab platform to optimize the predictive algorithms in order to prepare the tool in its final version.

The AI model will be re-trained with these new sub-score values and will furthermore be enriched with information about any fall accidents during and just before the 12 week intervention these results will be used by the DigiRehab platform to optimize the predictive algorithms in order to prepare the tool in its final version.

For the purpose of the usability assessment, through the administration of the SUS scale, an industry standard commonly used to assess the ease of user interaction with a digital system, it is expected to obtain indications of how the use of the app was evaluated by the users. This evaluation will be useful in order to identify any changes likely to improve the intervention.

A semi-structured satisfaction questionnaire will also be administered, which will aim to assess how well the intervention has been received by the target population and the extent to which it met their needs and those of the family caregiver (see Annex No. 5).

## Discussion and conclusion

Functional limitations in ADL are associated with risk of falling. Climbing or descending stairs (51%) and housekeeping (17%) are the most common indoor activities that led to head injury after falling in the older adults (12). Poor nutrition in old age has been associated with greater loss of muscle mass and muscle strength leading to increased risk of falls (13). Insomnia may be associated with significant daytime dysfunction and contributes to the risk of falls and fractures in this population (14). Dizziness and mobility problems have also been considered among the major risk factors for falls in the older adults (15, 16). Fear of falling (FOF) is one of the most common public health problems, which can lead to loss of confidence, reduced physical and social activities, depression, loss of mobility, and increased risk of falls (17). Other aspects such as personal care, health perception, vitality, social functioning, emotional problems, and mental health have also been used to question older adults subjects about their risk of falls in previous studies (18).

Certainly a sample of 50 subjects is not large, but it is also true that is a pilot study, and as such, by definition aims to provide inspiration for a possible larger future study, which will include an adequate sample size.

### The DigiRehab platform development

The DGR started as a team of highly qualified physiotherapists and IT-developers, aiming to setup an AI-ML training platform for the digital rehabilitation of older adults—committing full R&D between 2014 and 2018. Software algorithms have been developed and pilot tests performed, thus achieving TRL 4 by 2015. Between 2015 and 2017, DGR has developed pilot projects in DK, reaching TRL 5–6. In fact, DGR observed a reduction of 59 min/week/citizen in the need for homecare assistance in Denmark for the first 5 projects which included 2,500 citizens that concluded the 12 weeks intensive training program. In a control group of 800 citizens (not using the DGR training platform but participating in general rehabilitation measures offered by the municipality) there was an increase of homecare assistance in 20 min/week/citizen during the same timeframe. The DGR training platform had been optimized, matured, and validated (TRL 7–8) and since 2018 has been commercialized in DK and NO (TRL 9). Moreover, leveraging from the SC-AAL Project (E-Life), DGR further developed and optimized an app to serve as the user interface for the older adults to get access to physical training. By 2020, the app was market deployed together with DGR training platform.

By 2018, another opportunity to enlarge the risk of falls assessment has been identified. Therefore, DGR started the AIR [AAS1] Project in early 2020, to validate components of a potential DSS tool that could evaluate the risk of falling while anchored in data collection from different sources. That has been reached in early 2021 and now, with respect to the overall vision, the adaptation/optimization of a matured DSS platform, to target the defined markets, is currently situated at TRL 5.

### Physical strength and risk of falling in the older adults

The choice of these exercises follows the literature perspective on the main causes of falls in the older adults population. The 30 s sit to stand test has been already suggested by the “American Centre for Disease Control and Prevention” to predict patients’ risk of falls (19). The lunge test is reminiscent of the test developed by Wagenaar in 2012 as a predictive test for falls (20). The 4-m gait speed test as described is used by British Columbia government clinical protocols to detect frailty and falls in the older adults (21). The Timed up and go test has already been used to assess fall risk in the older adults living in nursing homes (22). The Push away or “Wall press-up” is one of the exercises recommended by the British NHS for the older adults (23). This exercise, although not focused on the lower limbs, could be extremely important for fall detection as the latest research shows a correlation between upper limb strength and fear of falls in the older adults (2). The last test “Getting down an up with the support of a chair” recalls standing exercises from a lying position, which is included in the physical activity guidelines for the older adults of the UK National Health System (24).

### Potential risks, burdens, and benefits for participants

We expect that by using the personalized physical training program through the DigiRehab training app, participants will experience an overall improvement in physical condition, quality of life, and decreased risk of falling. This will also result in reduced need for health and home care.

Regarding potential risks, the exercise program developed as part of the study is already in use in some of the countries participating in the project (Denmark, Norway and The Netherlands), and its implementation has not shown any particular risks or difficulties. As a precautionary measure, however, participants are always advised to check with their GP to discuss any concerns with them, and to inform the GP and our team if they recognize a sudden worsening of symptoms. Finally, access to the platform and therefore to the exercises will only be possible for the caregiver, who will be provided with an account. Therefore, the physical activity will be carried out only in the presence of the caregiver, who will be specially trained by specialized personnel to assist the older adults person during the personalized program and especially informed about how to avoid possible risks in relation to the characteristics of the older adults user. We can conclude that considering a potential risk of falling, such an event can definitely be avoided by the presence of the caregiver while performing the exercises.

### Expected impact

*Older adults users*: We expect that the personalized training targeting the older adults will help make them stronger and more independent by improving their quality of life and reducing their need for assistance. We also expect an alleviation of fear of falling and an increased propensity for going out of the home with consequent positive impacts on personal relationship life.

*Secondary end-users* (caregivers, informal caregivers): we expect that caregivers will benefit from being able to provide a more efficient care service to each user as it is based on the specific rehabilitation needs of each patient and the level of risk assigned at the initial assessment stage. This will optimize the quality of care provided and reduce care needs in the long run. In addition, by improving the physical condition of the older adults, informal caregivers will also be able to reduce their care burden by decreasing the home care needs of their relatives.

## Ethics statement

The studies involving humans were approved by the Ethical Committee of the IRCCS INRCA (reference ID: CE-INRCA-22022). The studies were conducted in accordance with the local legislation and institutional requirements. The participants provided their written informed consent to participate in this study.

## Author contributions

FD’A: Writing – original draft, Writing – review & editing. MH: Software, Supervision, Writing – original draft. DCo: Investigation, Methodology, Writing – original draft. ARB: Supervision, Writing – review & editing. DCi: Visualization, Writing – review & editing. NH: Data curation, Formal analysis, Methodology, Writing – original draft. AB: Funding acquisition, Project administration, Resources, Writing – review & editing. CFP: Data curation, Formal analysis, Methodology, Software, Writing – review & editing. PF: Data curation, Formal analysis, Writing – review & editing. CG: Project administration, Supervision, Writing – original draft, Writing – review & editing.

## Footnotes

^1^ https://digirehab.dk/en/
